# Determination of Optimal Vitamin D Dosage in Children with Cholestasis

**DOI:** 10.1186/s12887-023-04113-y

**Published:** 2023-06-21

**Authors:** Sirada Chongthavornvasana, Chatmanee Lertudomphonwanit, Pat Mahachoklertwattana, Manassawee Korwutthikulrangsri

**Affiliations:** 1 Department of Pediatrics, Faculty of Medicine Ramathibodi Hospital, Mahidol University, 270 Rama VI Road, Bangkok, 10400 Thailand; 2 Division of Gastroenterology, Department of Pediatrics, Faculty of Medicine Ramathibodi Hospital, Mahidol University, Bangkok, Thailand; 3 Division of Endocrinology, Department of Pediatrics, Faculty of Medicine Ramathibodi Hospital, Mahidol University, Bangkok, Thailand

**Keywords:** Cholestasis, Vitamin D deficiency, Biliary atresia, High-dose vitamin D treatment

## Abstract

**Background:**

Vitamin D deficiency in patients with cholestasis is due to impaired intestinal vitamin D absorption, which results from decreased intestinal bile acid concentration. Patients with cholestasis usually do not achieve optimal vitamin D status when a treatment regimen for children without cholestasis is used. However, data on high-dose vitamin D treatment in patients with cholestasis are limited.

**Methods:**

This study is a prospective study that included pediatric patients with cholestasis (serum direct bilirubin > 1 mg/dL) who had vitamin D deficiency (serum 25-hydroxyvitamin D, 25-OHD, < 20 ng/mL). In Phase 1, single-day oral loading of 300,000 IU (or 600,000 IU if weight ≥ 20 kg) of vitamin D2 was administered, followed by an additional loading if serum 25-OHD < 30 ng/mL, and 4-week continuation of treatment using a vitamin D2 dose calculated based on the increment of 25-OHD after first loading. In Phase 2, oral vitamin D2 (200,000 IU/day) was administered for 12 days, followed by 400,000 IU/day of vitamin D2 orally for another 8 weeks if serum 25-OHD < 30 ng/mL.

**Results:**

Phase 1: Seven patients were enrolled. Three out of seven patients had a moderate increase in serum 25-OHD after loading (up to 20.3–27.2 ng/mL). These patients had conditions with partially preserved bile flow. The remaining four patients, who had biliary atresia with failed or no Kasai operation, had low increments of serum 25-OHD. Phase 2: Eleven patients were enrolled. Eight out of 11 patients had a moderate increase in serum 25-OHD after 200,000 IU/day of vitamin D2 for 12 days. Serum 25-OHD continued increasing after administering 400,000 IU/day of vitamin D2 for another 8 weeks, with maximal serum 25-OHD of 15.7–22.8 ng/mL.

**Conclusion:**

Very high doses of vitamin D2 (200,000 and 400,000 IU/day) partly overcame poor intestinal vitamin D absorption and resulted in moderate increases in serum 25-OHD in pediatric patients with cholestasis, particularly when cholestasis was caused by uncorrectable bile duct obstruction.

## Background

Cholestasis, the impairment of bile flow caused by either hepatocellular dysfunction or biliary obstruction, is a common feature of liver diseases in children. Clinically, cholestasis is diagnosed when the conjugated or direct serum bilirubin level > 1 mg/dL when the total bilirubin is < 5 mg/dL, or when it is > 20% of the total bilirubin when the total bilirubin is > 5 mg/dL. Common identifiable causes of cholestasis during infancy include biliary atresia, congenital infection, hypopituitarism, and biliary sludge. In addition, various genetic and metabolic diseases that cause cholestasis, such as α1-antitrypsin deficiency, Alagille syndrome, and progressive familial intrahepatic cholestasis (PFIC), become more discovered [1]. While causes of chronic cholestasis in older children include choledochal cysts, cholelithiasis or choledocholithiasis, and inherited or inflammatory cholangiopathies [2–6]. The consequences of chronic cholestasis are hepatotoxicity resulting in cirrhosis and portal hypertension, pruritus, fat and fat-soluble vitamin malabsorption resulting in growth retardation and fat-soluble vitamin deficiency [7, 8].

Vitamin D is a fat-soluble hormone that is vital to children’s overall health. Vitamin D is essential for maintaining healthy bones because it regulates calcium and phosphorus metabolism. In addition, non-skeletal effects of vitamin D are possible in the prevention of several pathological conditions, including infectious and autoimmune diseases [9]. Therefore, vitamin D deficiency is related not only to musculoskeletal health but also to a wide range of acute and chronic diseases [10]. Vitamin D deficiency in patients with cholestasis is associated with intraluminal bile acid deficiency, which impairs vitamin D absorption [11, 12]. Moreover, in some patients with severe hepatic impairment, hepatic conversion of vitamin D to 25-hydroxyvitamin D (25-OHD) is also impaired [13].

The North American Society for Pediatric Gastroenterology, Hepatology and Nutrition and the European Society for Pediatric Gastroenterology Hepatology and Nutrition [14] recommend vitamin D supplementation for pediatric patients with cholestasis with dose adjustment according to serum 25-OHD levels. The optimal vitamin D doses for each patient vary depending on the degree of intestinal malabsorption. However, in studies of patients with cholestasis, most patients did not achieve optimal serum 25-OHD levels despite receiving a regular treatment regimen [15–17]. Thus, a higher dose of vitamin D treatment than given in previous studies may be required. In clinical practice, gradual dose titration usually takes several months. This may cause detrimental effects on the patient’s overall health, including impaired bone mass acquisition, rickets, and immune, cardiovascular, and respiratory system dysfunction [9]. Several previous studies reported that the severity of vitamin D deficiency in patients with cholestasis was associated with poor clinical outcomes, including incomplete response to ursodeoxycholic acid, cirrhosis development, liver-related mortality, and the need for liver transplantation [18–22].

The data regarding vitamin D treatment in pediatric patients with cholestasis are limited, especially treatment with high-dose vitamin D. This study aimed to determine the optimal regimen of vitamin D that would effectively increase serum 25-OHD level in pediatric patients with cholestasis.

## Methods

### Patient recruitment and characteristics

This was a prospective study conducted at the Department of Pediatrics, Faculty of Medicine, Ramathibodi Hospital, between April 2021 and August 2022. Pediatric patients (age 2 months–18 years) with cholestasis, which was defined as a direct/conjugated bilirubin value > 1 mg/dL continuing for more than 1 month, and vitamin D deficiency (serum 25-OHD level of less than 20 ng/mL) were enrolled in this study. Patients with ongoing improvement of cholestasis, such as resolving neonatal hepatitis, were excluded. The primary outcome was a serum 25-OHD level after completing the vitamin D treatment regimen.

Demographic and disease-related clinical data, including age, sex, weight, height, primary liver disease, previous surgical and medical treatment, and current medications, were collected. Disease severity was assessed using Pediatric End-Stage Liver Disease (PELD) score [23], in which higher scores indicate more severe disease. Biochemical parameters, liver function tests, coagulogram, and serum vitamin A, vitamin E, calcium, phosphate, and parathyroid hormone levels were collected at the beginning of the study.

The study was approved by the Ethics Committee of the Faculty of Medicine, Ramathibodi Hospital, Mahidol University (MURA 2021/256) on March 29, 2021. Written informed assent and consent were obtained from the patients and their legal guardians, respectively.

### Vitamin D treatment regimen and measurement of serum 25-OHD level

In this study, serum 25-OHD level was measured using the liquid chromatography-tandem mass spectrometry (LC-MS/MS) method (Agilent Triple Quadrupole LC/MS, United States). The preparation of vitamin D used in the study was a capsule containing 20,000 IU of vitamin D2. The whole capsules were administered if the patient was able to swallow them. For infants and patients who were unable to swallow capsules, the caregivers were instructed to open the capsule and dissolve the powder in a small amount of water.

#### Phase 1

Serum 25-OHD level was measured at baseline. Then, a single oral dose of vitamin D2 was administered (300,000 IU for the patients who weighed < 20 kg and 600,000 IU for those who weighed ≥ 20 kg) [24]. Serum 25-OHD level was measured 1 week later. If the patient’s serum 25-OHD level at 1 week was less than 30 ng/mL, suggestive of severely impaired intestinal vitamin D absorption, the additional (second) dose of vitamin D2 was administered within 4 days (1,200,000 IU for patients weighing < 20 kg and 2,400,000 IU for those weighing ≥ 20 kg). After the additional dose, serum 25-OHD level was measured 1 week later. The difference in serum 25-OHD levels between week 1 and baseline (Δ25-OHD_first_) was calculated.

The patients received daily oral vitamin D2 from the second week as a continuation treatment for 4 weeks. The daily dose of vitamin D2 was calculated using the following formula: (expected Δ25-OHD in children without cholestasis given the same high vitamin D dose / the patient’s actual Δ25-OHD_first_) x estimated daily dose for children without cholestasis. Serum 25-OHD level was measured again at the end of the continuation treatment.

The expected Δ25-OHD in children without cholestasis and estimated daily dose for children without cholestasis used in the calculation were based on previously reported values [24, 25] and adjusted to be approximate numbers as follows: 50 ng/mL and 2,000 IU/day, respectively for patients weighing < 10 kg; 40 ng/mL and 2,000 IU/day, respectively for patients weighing 10–19.9 kg; 50 ng/mL and 4,000 IU/day, respectively for patients weighing 20–39.9 kg; 30 ng/mL and 6,000 IU/day, respectively for patients weighing ≥ 40 kg. If the patient’s Δ25-OHD_first_ was < 0.5 ng/mL, the number of 0.5 ng/mL was used for the calculation.

### Phase 2

The treatment regimen was amended after the preliminary results of Phase 1 were collected and assessed. A higher dose and longer duration of vitamin D2 were used in Phase 2. Serum 25-OHD level was measured at baseline. Oral vitamin D2 dose of 200,000 IU/day was administered for 12 days, with a total dose of 2,400,000 IU over the administration period. We proposed this total dose based on the findings from Phase 1. The total dose of 2,400,000 IU was double the dose of 1,200,000 IU, which was used in the second loading in Phase 1. In addition, vitamin D2 administration was divided into multiple daily doses over several days instead of a single dose, with speculation of better intestinal vitamin D absorption. Serum 25-OHD level was measured 2 weeks after treatment. If the serum 25-OHD level was less than 30 ng/mL, 400,000 IU/day of oral vitamin D2, which was double the amount given in the Phase 2 daily dose, was administered daily for another 8 weeks. Serum 25-OHD level was measured again at weeks 4 and 8.

### Data analysis

Descriptive statistics were used in this study. Data were reported as the number (frequency) of patients for each particular result. Continuous variables were presented as median and range.

## Results

### Phase 1

**Fig. 1:**
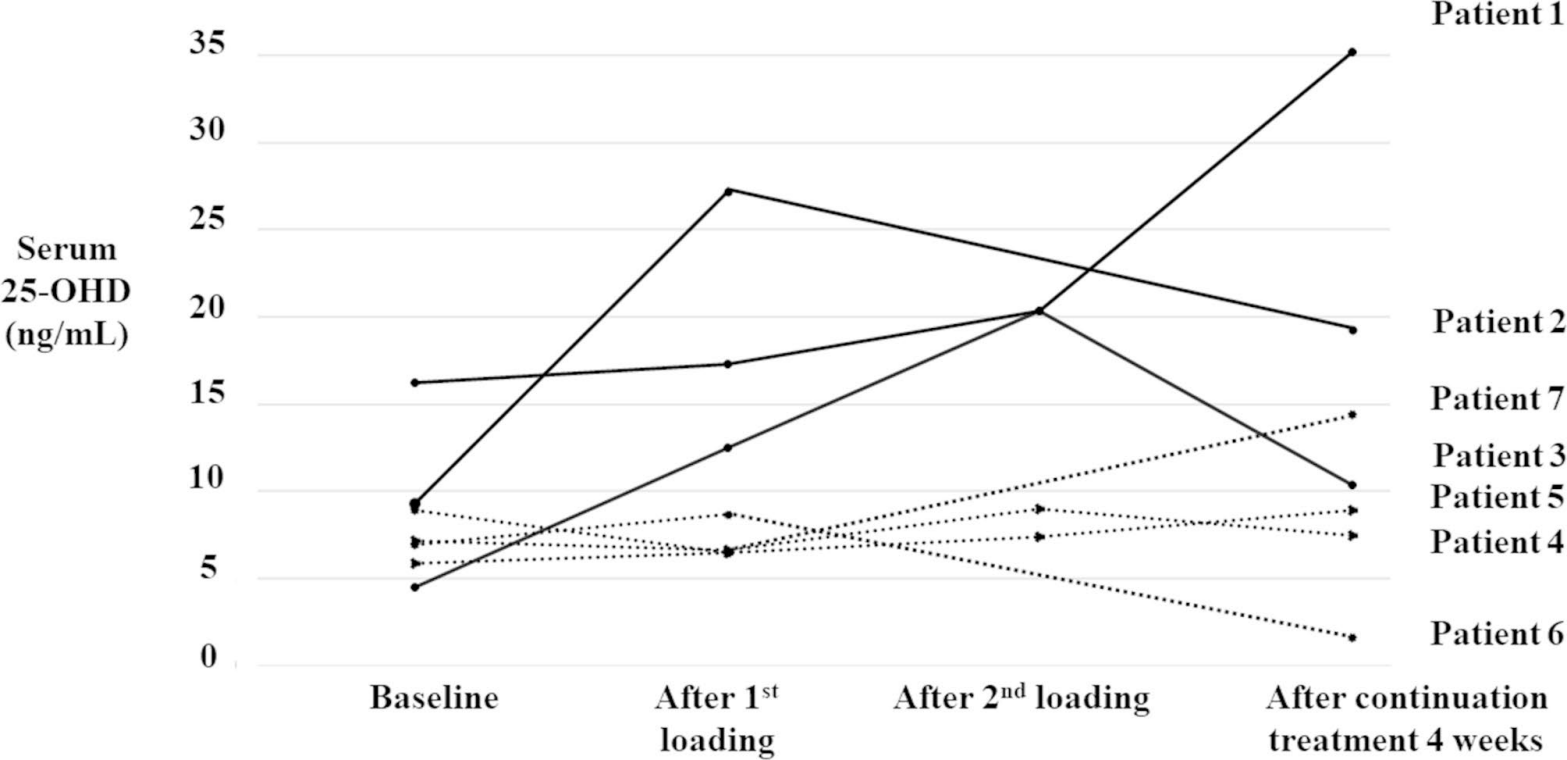
Serum 25-hydroxyvitamin D (25-OHD) level at baseline and after vitamin D2 treatment in Phase 1. Seven patients were enrolled. The graph shows serum 25-OHD levels after the first vitamin D2 loading (300,000 IU if weight < 20 kg or 600,000 IU if weight ≥ 20 kg), second loading (1,200,000 IU if weight < 20 kg or 2,400,000 IU if weight ≥ 20 kg), and after continuation treatment. Each line represents a patient

Seven patients were enrolled. The baseline clinical characteristics and biochemistry profile of the patients are shown in Table 1. The median (range) age of the patient was 1.4 (0.5–7.9) years. The median (range) direct bilirubin level was 13.4 (9.7–20.1) mg/dL. Serum 25-OHD levels at baseline and after vitamin D2 treatment are shown in Fig. 1. Three out of seven patients (Patients 1–3) had a moderate increase in serum 25-OHD level, with maximal serum 25-OHD level at any time point after treatment of > 20 ng/mL (range 20.3–27.2 ng/mL). The difference in serum 25-OHD levels between maximal and baseline (Δ25-OHD_max_) was 19.0, 17.9, and 15.8 ng/mL in patient 1, 2, and 3, respectively. These patients had conditions with partially preserved bile flow. Patient 1, who had biliary atresia, had already undergone liver transplantation but still had cholestasis due to post-operative bile duct anastomosis obstruction. Patient 2, who had biliary atresia, previously had a successful Kasai operation but had cholestasis due to decompensated cirrhosis, which developed over time. Patient 3 had Alagille syndrome, in which cholestasis is caused by intrahepatic bile duct paucity, not by anatomical obstruction. Serum 25-OHD levels of patients 2 and 3 decreased after the continuation of treatment using a calculated dose from the proposed formula based on their Δ25-OHD_first_. The serum 25-OHD level of patient 1 increased after the continuation of treatment. Notably, however, patient 1 underwent surgical intervention to correct bile duct anastomosis obstruction during the continuation treatment period.

**Table 1:** Baseline clinical characteristics and biochemical profiles of the enrolled patients in Phase 1

Patient	Diagnosis	Remarks	Age (years)	Weight (kg)	TB (mg/dL)	DB (mg/dL)	GGT (U/L)	Alb (g/L)	INR		PELD score	Calcium (mg/dL)	Phosphate (mg/dL)	PTH (pg/mL)	Vitamin A (mg/L)	Vitamin E (mg/g cholesterol)
1	BA	Post LT, bile duct anastomosis obstruction	7.9	21.0	9.9	7.6	468	29		1.3	15	8.6	3.9	22	0.4	2.4
2	BA	Successful Kasai operation, decompensated cirrhosis	7.8	22.9	13.0	9.7	200	26		1.3	17	8.7	5.0	28	0.2	4.8
3	Alagille syndrome	Intrahepatic bile duct paucity	1.7	8.1	23.3	17.0	632	30		1.0	12	10.2	5.2	46	0.3	0.0
4	BA	No Kasai operation	0.8	8.9	21.2	14.9	45	18		1.8	26	7.8	3.8	34	0.1	0.5
5	BA	Failed Kasai operation	1.4	8.3	27.2	20.1	253	28		1.8	20	9.4	4.2	37	0.2	0.5
6	BA	No Kasai operation	0.6	6.5	13.4	9.7	507	23		1.3	16	8.2	4.0	22	0.1	1.3
7	BA	No Kasai operation	0.5	6.8	18.9	13.4	775	26		1.2	15	9.1	3.5	27	0.1	0.3

Alb, albumin; BA, biliary atresia; DB, direct bilirubin; GGT, gamma-glutamyl transferase; INR, the international normalized ratio;

LT, liver transplantation; PELD, Pediatric End-Stage Liver Disease; PTH, parathyroid hormone; TB, total bilirubin

The remaining four out of seven patients (patients 4–7) had minimal or no increase in serum 25-OHD level after vitamin D treatment. These patients had biliary atresia and either had a failed Kasai operation (defined as serum total bilirubin > 6 mg/dL at 3 months after surgery) [26] or had never undergone a Kasai operation. Maximal serum 25-OHD levels of patients 4, 5, 6, and 7 after vitamin D2 treatment were 9, 8.9, 8.7, and 14.4 ng/mL, respectively, which were still in the range of deficiency (< 20 ng/mL). The Δ25-OHD_max_ of patients 4, 5, 6, and 7 were 1.8, 3.0, 1.8, and 5.5 ng/mL, respectively. Median (range) ages were 7.8 (1.7–7.9) vs. 0.7 (0.5–1.4) years in the group with moderate and minimal/no response, respectively. Median (range) direct bilirubin levels were 9.7 (7.6–17.0) vs. 14.2 (9.7–20.1) mg/dL in the moderate and minimal/no response groups, respectively.

#### Phase 2

**Fig. 2:**
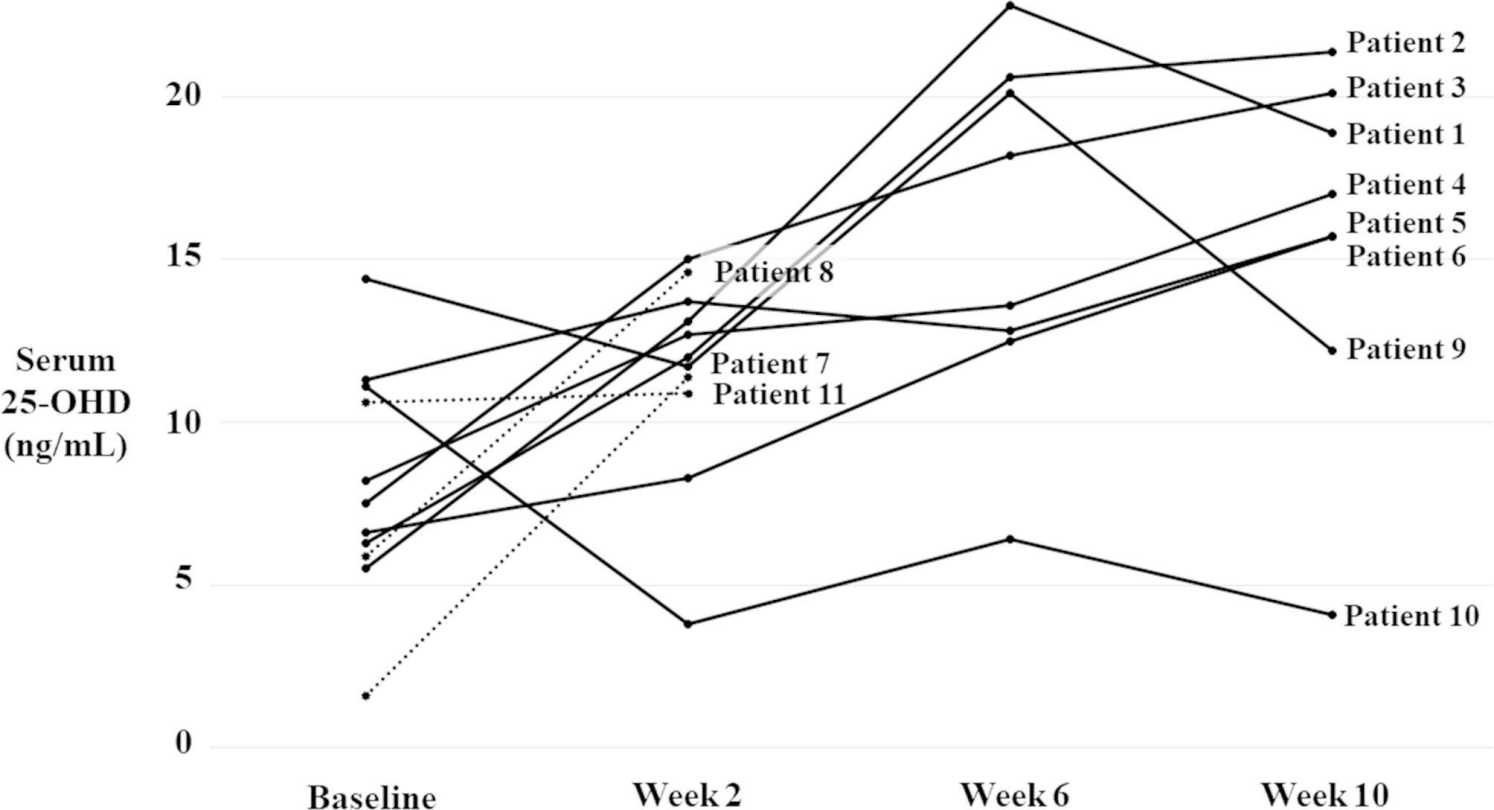
Serum 25-hydroxyvitamin D (25-OHD) level at baseline and after vitamin D2 treatment in Phase 2. Eleven patients were enrolled. All patients had biliary atresia. The graph shows serum 25-OHD levels at week 2 (200,000 IU/day for 12 days), week 6 (400,000 IU/day), and week 10 (400,000 IU/day) after treatment. Each line represents a patient

Eleven patients were enrolled. All the patients in this phase had biliary atresia, and they had either had failed Kasai operations or had never undergone a Kasai operation. Baseline clinical characteristics and biochemistry profiles of the patients are shown in Table 2. The median (range) age of the patients was 0.8 (0.5–1.8) years. The median (range) direct bilirubin level was 11.5 (8.9–18.5) mg/dL. Three patients (patients 7, 8, and 11) dropped out during the study because of liver transplantation, loss to follow-up, and death due to COVID-19 infection, respectively. Serum 25-OHD levels at baseline and after vitamin D2 treatment are shown in Fig. 2.

**Table 2:** Baseline clinical characteristics and biochemical profiles of the enrolled patients in Phase 2

Patient*	Age (years)	Weight (kg)	TB (mg/dL)	DB (mg/dL)	GGT (U/L)	Alb (g/L)	INR	PELD score	Calcium (mg/dL)	Phosphate (mg/dL)	PTH (pg/mL)	Vitamin A (mg/L)	Vitamin E (mg/g cholesterol)
1	0.9	10.3	18.6	13.6	367	25	1.3	24	8.8	4.2	43	0.2	0.9
2	0.8	8.5	15.5	11.5	246	29	1.0	17	8.4	4.3	22	0.2	0.0
3	1.8	13.0	20.0	14.6	466	20	1.3	14	8.5	3.9	14	0.1	1.4
4	0.5	7.1	13.2	9.4	543	19	1.3	24	8.8	3.9	24	0.1	1.1
5	0.8	7.8	13.1	9.5	1930	27	1.0	17	10.0	4.9	33	0.3	0.3
6	1.6	8.9	26.9	18.5	604	31	1.1	16	9.1	4.6	34	0.2	0.5
7	0.6	7.0	14.9	10.7	336	20	1.3	24	7.9	4.2	19	0.1	1.1
8	0.5	6.4	12.4	8.9	884	24	1.2	20	8.9	4.1	56	0.1	0.9
9	0.7	6.9	15.1	10.9	383	26	1.3	22	8.4	3.9	34	0.1	0.4
10	0.5	6.5	18.5	13.5	335	25	1.2	15	9.6	3.8	28	0.0	0.6
11	0.8	6.7	18.3	12.9	53	24	1.3	24	8.4	4.2	25	0.2	1.5

*All of the patients in Phase 2 had biliary atresia and either had failed Kasai operation or never underwent Kasai operation and had not undergone liver transplantation

Alb, albumin; BA, biliary atresia; DB, direct bilirubin; GGT, gamma-glutamyl transferase; INR, the international normalized ratio;

LT, liver transplantation; PELD, Pediatric End-Stage Liver Disease; PTH, parathyroid hormone; TB, total bilirubin

After 200,000 IU/day of vitamin D2 for 12 days, Patients 1–8 had a moderate increase in serum 25-OHD level. The median (range) serum 25-OHD level at week 2 was 12.9 (8.3–15.0) ng/mL. Median (range) difference between 2-week post-treatment and baseline serum 25-OHD was 6.6 (1.7–9.8) ng/mL. Serum 25-OHD continued increasing after administering the 400,000 IU/day dose of vitamin D2 for 8 weeks. The median (range) maximal serum 25-OHD level at any time point after treatment was 16.4 (11.4–22.8) ng/mL. Median (range) Δ25-OHD_max_ was 9.5 (4.4–17.3) ng/mL. Patients 9–11 had minimal or no increase in serum 25-OHD levels after 200,000 IU/day of vitamin D2 for 12 days. After the dose of 400,000 IU/day of vitamin D2 was administered, patient 9 had an increase in serum 25-OHD level to 20.1 ng/mL (Δ25-OHD_max_ 5.7 ng/mL). Patient 10 had low serum 25-OHD level at every time point after treatment without any increase. Patient 11 had no follow-up due to death caused by COVID-19 infection.

## Discussion

Patients with cholestasis have impaired intestinal vitamin D absorption, and thus have difficulty achieving optimal serum 25-OHD levels. In this study, we demonstrated that higher doses and longer duration of vitamin D treatment, compared with the general recommendation, can partially improve the increment of serum 25-OHD levels in pediatric patients with cholestasis. Patients with uncorrectable anatomical obstruction of the bile duct had the most difficulty achieving the optimal desired serum 25-OHD level.

In Phase 1, infants with biliary atresia who had never undergone or had a failed Kasai operation had poor responses to oral single-day loading of 300,000 IU of vitamin D2 and a higher loading dose of 1,200,000 IU. The findings are suggestive of poor intestinal vitamin D absorption, which is likely caused by a deficiency of intestinal bile acid in these patients due to severe anatomical obstruction of the bile duct. Single-day loading might also contribute to poorer absorption than divided multiple daily doses given over several days.

Although single high-dose vitamin D therapy (stoss therapy) was previously reported to be effective for treating of vitamin D deficiency in infants and young children without cholestasis [27], it did not result in an optimal increment of serum 25-OHD levels in infants with cholestasis in this study. A previous study reported treatment with oral high-dose vitamin D2 of 300,000 IU over 2–3 days in four infants with cholestasis. Four weeks after treatment, none of these patients had achieved a serum 25-OHD level of more than 20 ng/mL [28]. This finding suggested that even though a high-dose vitamin D treatment was used, it was still inadequate for this group of patients, which agrees with the finding in most of our patients in the Phase 1 study.

Despite these results, some patients in Phase 1 had a moderate increase in serum 25-OHD levels after high-dose vitamin D treatment. Their clinical characteristics were different from the group of patients without response to treatment. They had biliary atresia post-liver transplantation, post-successful Kasai operation, or Alagille syndrome. Although cholestasis was present in these patients, intestinal bile flow might be more preserved than in the other biliary atresia patients with a failed Kasai operation. Despite a moderate increase in serum 25-OHD level after loading, serum 25-OHD levels of patients 2 and 3 decreased after the continuation treatment, suggesting that dose calculation using Δ25-OHD_first_ in the proposed formula underestimated the required dose. Further studies are needed to determine the dose for continuation treatment in this group of patients whose intestinal bile flow is partially preserved.

To achieve optimal serum 25-OHD level, Phase 2 was conducted using an adjusted treatment protocol. A total of 2,400,000 IU of vitamin D2 was given in multiple daily doses over 2 weeks, and it resulted in a higher proportion of patients with a moderate increase in serum 25-OHD level (8 out of 11 infants with biliary atresia). Continuation of treatment with increment dosage resulted in a further increase in serum 25-OHD levels, though the sufficient level of 30 ng/mL was not reached. The finding of a higher increase in serum 25-OHD levels in Phase 2, in which higher doses and longer duration of vitamin D was commenced, compared with Phase 1 suggested that the major factor of low serum 25-OHD levels in patients with cholestasis is impaired intestinal vitamin D absorption, rather than a defect in hepatic 25-hydroxylation. A higher daily dose given over a longer period could partially overcome the absorption defect and result in a higher level of serum 25-OHD. Another previous study using high doses of vitamin D therapy (vitamin D2 50,000 IU, three times a week over 10 weeks) led to the improvement of serum 25-OHD levels and resolution of rickets in PFIC type 2. [29] The pathogenesis of cholestasis in PFIC type 2, which is associated with impaired function of hepatocyte bile salt export pump [30], differs from that of biliary atresia, which is mainly caused by anatomical bile duct obstruction. This may explain the better outcomes of vitamin D treatment in patients with PFIC type 2 compared with the present study. In addition, giving multiple daily doses of vitamin D2 over several days instead of a single dose may be more effective because these patients might have limited absorption per dose due to limited bile flow in each period.

Previous studies have also reported on vitamin D supplementation in the form of multiple fat-soluble vitamins (FSV) mixed with tocopheryl polyethylene glycol-1000 succinate (TPGS) in children with cholestasis [31, 32]. TPGS has amphipathic properties, thus potentially enhancing absorption independent of intraluminal bile acid. In one study in infants with biliary atresia, 79% of infants with persistent cholestasis (total bilirubin level > 2 mg/dL) still had serum 25-OHD level < 15 ng/mL after 6-month supplementation with FSV/TPGS [31]. In another study, 60% of patients had serum 25-OHD level < 14 ng/mL after 3-month supplementation [32]. These results, as well as the data from the present study, suggest that developing an oral regimen that can effectively correct vitamin D deficiency in this group of patients is still challenging. Different routes of vitamin D administration that bypass intestinal absorption, such as the intramuscular route, is more beneficial for this group of patients. Unfortunately, the intramuscular form of vitamin D is unavailable in Thailand, and it may also be the same in some other countries. Therefore, high dose oral vitamin D treatment is still needed to treat these patients.

The strength of this study is the implementation of the higher dose of vitamin D treatment in children with cholestasis to facilitate the desired serum 25-OHD levels, especially because similar studies in this population are limited. Furthermore, we used liquid chromatography-mass spectrometry (LC-MS/MS) to measure 25-OHD levels, which is the most reliable method [33].

We acknowledge that this study had limitations. First, requiring a large amount of vitamin D capsules could cause difficulty for patients and families in clinical practice. Second, this study was a single-center design study with a small number of patients. Further studies in multiple centers and larger groups of children with various degrees of cholestasis are needed.

## Conclusions

The findings of this study suggest that very high multiple daily doses of vitamin D2 treatment may result in a better increment of serum 25-OHD level in children with cholestasis, particularly in cases of cholestasis caused by bile duct obstruction.

## Data Availability

The datasets generated and analyzed during the current study are not publicly available because sharing could compromise individual privacy. Data are available from the corresponding author upon reasonable request.

## Abbreviations

25-OHD: 25-Hydroxyvitamin D
Δ25-OHD_first_: The difference in serum 25-hydroxyvitamin D level between week 1 (after the first dose of vitamin D treatment) and baseline
Δ25-OHD_max_: The difference in serum 25-hydroxyvitamin D level between maximal level at any time point and baseline
FSV: Fat-soluble vitamin
LC-MS/MS: Liquid chromatography-tandem mass spectrometry
PELD: Pediatric End-Stage Liver Disease
PFIC: Progressive familial intrahepatic cholestasis
TPGS: Tocopheryl polyethylene glycol-1000 succinate

## References

[1] Feldman AG, Sokol RJ (2019). Neonatal cholestasis: emerging molecular diagnostics and potential novel therapeutics. Nat Reviews Gastroenterol Hepatol.

[2] Karpen SJ (2020). Pediatric cholestasis: epidemiology, genetics, diagnosis, and current management. Clin Liver Disease.

[3] Pogorelic Z, Aralica M, Jukic M, Zitko V, Despot R, Juric I (2019). Gallbladder disease in children: a 20-year single-center experience. Indian Pediatr.

[4] Pogorelic Z, Lovric M, Jukic M, Perko Z (2022). The laparoscopic cholecystectomy and common bile Duct Exploration: a single-step treatment of Pediatric Cholelithiasis and Choledocholithiasis. Children.

[5] Uchida H, Tiao G, Shivakumar P, Wong K, Asai A, Amano H. Infants with cholestasis.Front Pead.2023;11. 10.3389/fped.2023.1175231 PMC10086434 37056945

[6] Kowalski A, Kowalewski G, Kalicinski P, Pankowska-Wozniak K, Szymczak M, Ismail H, Stefanowicz M (2023). Choledochal cyst excision in infants—A retrospective study. Children.

[7] Assis DN (2018). Chronic complications of cholestasis: evaluation and management. Clin Liver Dis.

[8] Patil A, Mayo MJ, Lindor KD, Talwalkar JA (2008). Complications of Cholestasis. Cholestatic Liver Disease. Clinical gastroenterology.

[9] Saggese G, Vierucci F, Boot AM, Czech-Kowalska J, Weber G, Camargo CA (2015). Vitamin D in childhood and adolescence: an expert position statement. Eur J Pediatr.

[10] Misra M, Pacaud D, Petryk A, Collett-Solberg PF, Kappy M (2008). Drug and therapeutics Committee of the Lawson Wilkins Pediatric Endocrine Society. Vitamin D deficiency in children and its management: review of current knowledge and recommendations. Pediatrics.

[11] Pappa HM, Bern E, Kamin D, Grand RJ (2008). Vitamin D status in gastrointestinal and liver disease. Curr Opin Gastroenterol.

[12] Kamath BM, Alonso EM, Heubi JE, Karpen SJ, Sundaram SS, Shneider BL (2022). Fat Soluble vitamin Assessment and Supplementation in Cholestasis. Clin Liver Dis.

[13] Capul EP, Gregorio GEV, Pauig JP (2022). Relationship of serum vitamin D with liver disease severity and bone abnormalities in cholestatic children. Acta Med Philippina.

[14] Mouzaki M, Bronsky J, Gupte G, Hojsak I, Jahnel J, Pai N (2019). Nutrition support of children with chronic liver diseases: a joint position paper of the North American Society for Pediatric Gastroenterology, Hepatology, and Nutrition and the european Society for Pediatric Gastroenterology, Hepatology, and Nutrition. J Pediatr Gastroenterol Nutr.

[15] Ng J, Paul A, Wright N, Hadzic N, Davenport M (2016). Vitamin D levels in infants with biliary atresia: pre-and post-kasai portoenterostomy. J Pediatr Gastroenterol Nutr.

[16] Anwar MM, Arafa AE, Morgan DS, Mohamed KK (2018). Association between vitamin D level and patients with cholestasis. Int J Community Med Public Health.

[17] da Rocha CRM, Kieling CO, Adami MR, Guedes RR, Guaragna Filho G, Vieira SMG (2021). Evaluation of the response to treatment of vitamin D deficiency in pediatric patients with chronic liver disease. Ann Hepatol.

[18] Ebadi M, Rider E, Tsai C, Wang S, Lytvyak E, Mason A (2023). Prognostic significance of severe vitamin D Deficiency in patients with primary sclerosing Cholangitis. Nutrients.

[19] Ebadi M, Ip S, Lytvyak E, Asghari S, Rider E, Mason A (2022). Vitamin D is associated with clinical outcomes in patients with primary biliary cholangitis. Nutrients.

[20] Wang Z, Peng C, Wang P, Sui J, Wang Y, Sun G (2020). Serum vitamin D level is related to disease progression in primary biliary cholangitis. Scand J Gastroenterol.

[21] Guo GY, Shi YQ, Wang L, Ren X, Han ZY, Guo CC (2015). Serum vitamin D level is associated with disease severity and response to ursodeoxycholic acid in primary biliary cirrhosis. Aliment Pharmacol Ther.

[22] Zhuang P, Sun S, Dong R, Chen G, Huang Y, Zheng S. Associations between Vitamin D and liver function and liver fibrosis in patients with biliary atresia.Gastroenterology Research and Practice.2019;2019, 4621372. 10.1155/2019/4621372 PMC6875370 31781188

[23] Bourdeaux C, Tri TT, Gras J, Sokal E, Otte J-B, De Goyet JDV (2005). PELD score and posttransplant outcome in pediatric liver transplantation: a retrospective study of 100 recipients. Transplantation.

[24] McNally JD, Iliriani K, Pojsupap S, Sampson M, O’Hearn K, McIntyre L (2015). Rapid normalization of vitamin D levels: a meta-analysis. Pediatrics.

[25] Munns CF, Shaw N, Kiely M, Specker BL, Thacher TD, Ozono K (2016). Global consensus recommendations on prevention and management of nutritional rickets. Hormone Res paediatrics.

[26] Davenport M, Heaton N, Superina R, editors. (2017).Surgery of the Liver, Bile Ducts and Pancreas in Children (3rd ed.). London:CRC Press; 2017.p.71–86.

[27] Shah BR, Finberg L (1994). Single-day therapy for nutritional vitamin D-deficiency rickets: a preferred method. J Pediatr.

[28] Jensen M, Abu-El-Haija M, Bishop W, Rahhal RM (2015). Difficulty achieving vitamin D sufficiency with high-dose oral repletion therapy in infants with cholestasis. J Pediatr Gastroenterol Nutr.

[29] Sura SR, Germain-Lee EL (2020). Treatment of rickets and dyslipidemia in twins with progressive familial intrahepatic cholestasis type 2. Int J Pediatr Endocrinol.

[30] Felzen A, Verkade HJ (2021). The spectrum of progressive familial intrahepatic cholestasis diseases: update on pathophysiology and emerging treatments. Eur J Med Genet.

[31] Shneider BL, Magee JC, Bezerra JA, Haber B, Karpen SJ, Raghunathan T (2012). Efficacy of fat-soluble vitamin supplementation in infants with biliary atresia. Pediatrics.

[32] Shen YM, Wu JF, Hsu HY, Ni YH, Chang MH, Liu YW (2012). Oral absorbable fat-soluble vitamin formulation in pediatric patients with cholestasis. J Pediatr Gastroenterol Nutr.

[33] Zelzer S, Goessler W, Herrmann M (2018). Measurement of vitamin D metabolites by mass spectrometry, an analytical challenge. J Lab Precis Med.

